# Comprehensive Analysis of the Profiles of Differentially Expressed mRNAs, lncRNAs, and circRNAs in Phosgene-Induced Acute Lung Injury

**DOI:** 10.1155/2021/6278526

**Published:** 2021-01-09

**Authors:** Yiru Shao, Zhifeng Jiang, Daikun He, Jie Shen

**Affiliations:** ^1^Center of Emergency & Intensive Care Unit, Jinshan Hospital, Fudan University, Shanghai 201508, China; ^2^Medical Center of Chemical Injury, Jinshan Hospital, Fudan University, Shanghai 201508, China; ^3^Medical Research Center for Chemical Injury, Emergency and Critical Care of Chemical Injury, Jinshan Hospital, Fudan University, Shanghai 201508, China

## Abstract

Phosgene exposure can cause acute lung injury (ALI), for which there is no currently available effective treatment. Mesenchymal stem cells (MSCs) which have been proven to have therapeutic potential and be helpful in the treatment of various diseases, but the mechanisms underlying the function of MSCs against phosgene-induced ALI are still poorly explored. In this study, we compared the expression profiles of mRNAs, lncRNAs, and circRNAs in the lung tissues from rats of three groups—air control (group A), phosgene-exposed (group B), and phosgene + MSCs (group C). The results showed that 389 mRNAs, 198 lncRNAs, and 56 circRNAs were differently expressed between groups A and B; 130 mRNAs, 107 lncRNAs, and 35 circRNAs between groups A and C; and 41 mRNAs, 88 lncRNAs, and 18 circRNAs between groups B and C. GO and KEGG analyses indicated that the differentially expressed RNAs were mainly involved in signal transduction, immune system processes, and cancers. In addition, we used a database to predict target microRNAs (miRNAs) interacting with circRNAs and the *R* network software package to construct a circRNA-targeted miRNA gene network map. Our study showed new insights into changes in the RNA expression in ALI, contributing to explore the mechanisms underlying the therapeutic potential of MSCs in phosgene-induced ALI.

## 1. Introduction

Phosgene is an indispensable mass production, used as intermediate in the manufacture of building blocks of various types of plastics, medicine, dye, and other chemical products [1]. It was reported that individuals accidentally exposed to phosgene at approximately >600 mg/m^3^×min developed clinically significant phosgene-induced ALI [2]. Short-term exposure to phosgene leading to ALI and prolonged exposure would cause the fatal acute respiratory distress syndrome [3–5]. Now, exploring the potential molecular therapeutic targets for phosgene-induced ALI is needed.

Systemically, administered mesenchymal stem cells (MSCs) have the ability to selectively target sites of tissue injury or inflammation [6]. Exogenously administered MSCs have been observed to ameliorate lung injury in various animal models, including endotoxin-induced ALI [7], lipopolysaccharide- (LPS-) induced lung injury [8], and phosgene-induced ALI [9]. Several studies have demonstrated that facilitation of MSC localization to injured tissue sites can incrementally benefit ALI [10, 11]. Thus, facilitation of MSC localization to target tissue sites represents a promising therapeutic strategy for ALI.

Most of the transcribed RNAs are identified as noncoding RNAs, which may be fully responsible for the complex gene expression in humans [12]. Recent years, mounting evidence has shown that lncRNAs and circRNAs play important roles in the regulation of the gene expression [13, 14]. lncRNAs are a new class of regulatory RNAs over 200 nucleotides in length, and circRNAs are a class of ncRNAs that have stable structures and are resistant to the absence of 5′ or 3′ ends [15, 16]. Emerging evidence suggests the involvement of lncRNAs and circRNAs in lung injury. For example, lncRNA TUG1 alleviates sepsis-induced ALI [17], whereas circANKRD36 silencing can alleviate LPS-irritated human embryonic lung fibroblast cell injury [18]. To date, the profiles of ncRNAs, particularly circRNAs, and their roles in phosgene-exposed ALI have not been completely elucidated.

In our study, we conducted transcriptome sequencing to determine the expression profiles of mRNAs, lncRNAs, and circRNAs in a phosgene-exposed ALI model. Furthermore, we conducted GO and KEGG analyses and build the circRNA-miRNA coexpression networks. Our study is aimed at elucidating the molecular mechanisms of phosgene-induced ALI after MSC treatment to identify biomarkers and new therapeutic targets for lung injury.

## 2. Materials and Methods

### 2.1. Experimental Animals and Sample Collection

All experimental procedures involving animals were approved by the Animal Care and Use Committee of Jinshan Hospital affiliated to Fudan University, China. A rat model of phosgene-induced ALI was constructed as described previously [5]. The rats were divided into three groups—air control (group A, *n* = 3), phosgene-exposed (group B, *n* = 3), and phosgene + MSCs (group C, *n* = 3). The rats in group A were exposed to normal room air, whereas the rats in the groups B and C were exposed to air comprising 8.33 mg/L phosgene for 5 min. Rats were intravenously injected with MSCs (10^6^ cells per rat) via the tail vein. The rats' lung tissues were analyzed to determine the degree of MSC localization after 48 h.

### 2.2. RNA Extraction, Library Construction, and Sequencing

Total RNA was obtained from the lung tissues with the mirVana miRNA Isolation Kit (Ambion, Inc. Austin, TX, USA) according to the manufacturer's instructions. RNA integrity was assessed by the Agilent 2100 Bioanalyzer (Agilent Technologies, USA). RNA libraries were established through TruSeq Stranded Total RNA with Ribo-Zero Gold (RS-122-2301, Illumina, San Diego, CA, USA) and then were sequenced on an Illumina sequencing platform (HiSeqTM 2500, Illumina, San Diego, CA), and 150 bp/125 bp paired-end reads were produced.

### 2.3. Reference Genome Mapping and Transcriptome Assembly

Raw reads generated during high-throughput sequencing were FASTQ format sequences. High quality reads obtained can be used for subsequent analysis, and these raw reads needed to be filtered further in terms of quality. Trimmomatic software was first used to remove adapters, after which low-quality bases, N-bases, and low-quality reads were filtered out. Finally, we obtained high-quality clean reads. The Q30 (*Q* score of 30) and GC contents of the clean data were then measured. The samples were assessed by genomic and gene alignment using HiSAT2 to align clean reads to the reference genome of the experimental species.

### 2.4. Identification of lncRNAs

Candidate lncRNA sets were subjected to the following rigorous screening steps for subsequent analysis. (1) The merged transcripts were compared with a known reference gene model using Cuffcompare software, and “*I*,” “*u*,” “*x*,” and “*o*” transcripts were retained. (2) Transcripts of >200 bp and with ≥2 exons were selected. (3) The obtained transcripts were predicted using CPC2, CNCI, PLEK, and Pfam databases, and the transcripts from the intersection of these four databases were screened to obtain candidate lncRNAs. (4) The predicted lncRNA sequences were compared to known lncRNA sequences through BLAST software and were thus identified as known lncRNAs. For species without known lncRNAs, the predicted lncRNA sequences obtained were directly used for quantitative analysis.

### 2.5. Identification of circRNAs

CIRI software is highly sensitive and can be used to perform multiple screenings to reduce false positives; therefore, it is an authoritative software for circRNA prediction. In the present study, the CIRI software was based on the new alignment algorithm BWA-MEM comparison results, and the specific prediction process was as follows: (1) SAM files were obtained by the BWA-MEM comparison of clean reads with the genome of the reference species. (2) Balanced junction reads were detected based on xS/HyM (upstream) or xMyS/H (downstream) paired chiastic clipping signals. (3) Junction reads were filtered based on paired-end mapping and GT/AG signals. (4) Junction reads were detected based on a DM algorithm. After CIRI prediction for each sample, a single circ-bed was obtained, and all samples were merged into the circ-bed. Then, the number of junction reads in different samples of each predicted circRNA was counted, and the reads per million of each circRNA were calculated.

### 2.6. Differential Screening Analysis and Functional Analysis

The estimate size factor function of the DESeq (2012) *R* package was performed to normalize the counts, and the nbinomTest function was carried out to statistical *P* values and fold change values to compare differences among transcripts. Differential transcripts with *P* values of ≤0.05 and a fold change of ≥2 were chosen, and all genes were mapped to GO terms through the GO analysis. Differential RNA GO (http://www.geneontology.org/) and KEGG (https://www.genome.jp) analyses were carried out through the hypergeometric distribution test.

### 2.7. CeRNA Network Construction

Based on differentially expressed circRNA data, we used a database to predict target miRNAs interacting with circRNAs. For the enrichment results of total differences in circRNAs, the top 300 miRNA–circRNA interaction pairs with small *P* values were extracted in order of the *P* value, and the *R* package network was performed to establish a circRNA-targeted miRNA network map.

## 3. Results

### 3.1. Summary of Raw Sequence Reads

After removing low-quality sequences, a total of 277.01, 288.96, and 283.3 million clean reads with Q30 (*Q* score of 30) of >91.55% were obtained from groups A, B, and C, respectively (Table 1). About 96% of the reads were aligned to the reference genome (Table 1).

### 3.2. The Profiles of Differentially Expressed of mRNAs, lncRNAs, and circRNAs

**Figure 1:**
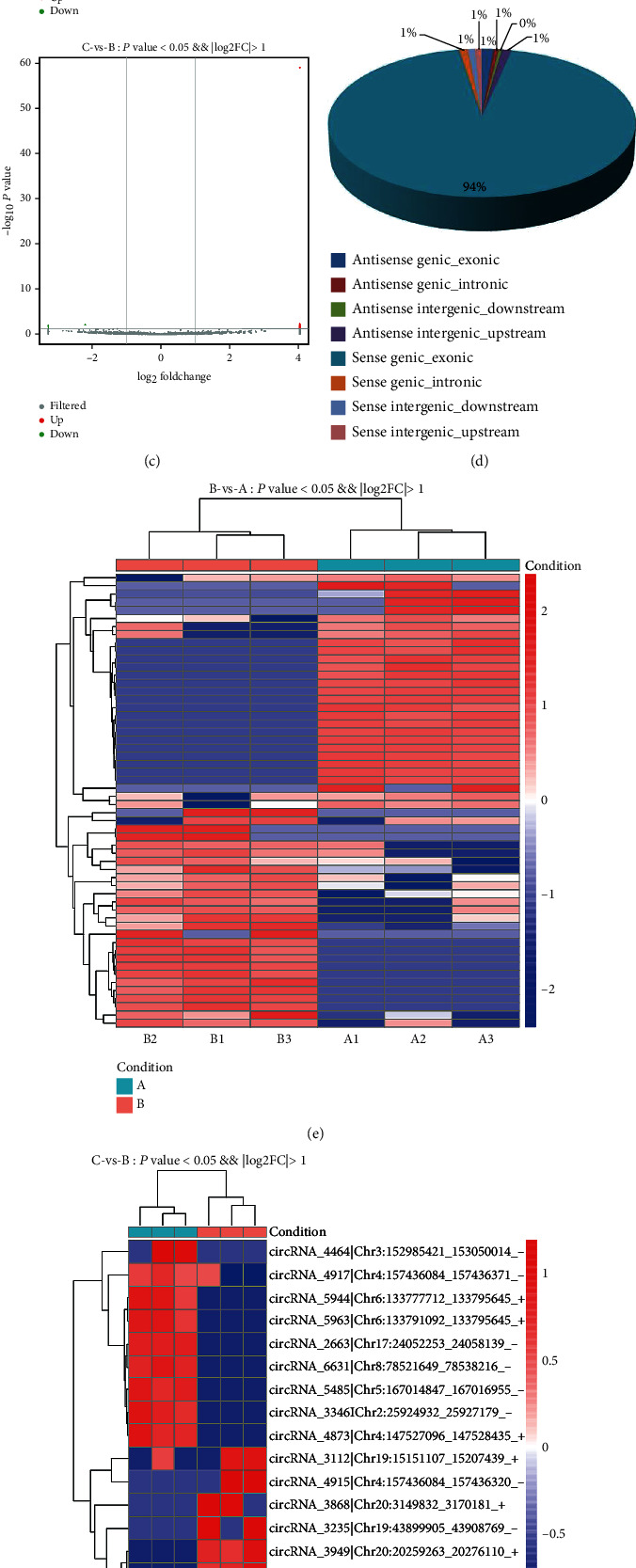
Differentially expressed circRNAs in the lung tissues of three groups of rats. (a) A box and whisker plot of circRNAs showing the distribution of RNA intensities in all samples. (b) Heat maps of correlation coefficients for all samples. (c) Volcano plots showing variation in the circRNA expression. The vertical lines correspond to the 2-fold change, and the horizontal line represents a *P* value of 0.05. (d) Type and proportion of circRNAs. (e) Hierarchical clustering of all differentially expressed circRNAs in the lung tissues of rats in groups A and B. (f) Hierarchical clustering of all differentially expressed circRNAs in the lung tissues of rats in groups B and C. (g) Differentially expressed genes were analyzed using DEGseq software based on the fragments per kilobase of the transcript per million mapped reads (FPKM) method (≥2-fold change with *P* <0.05). The number of differentially expressed circRNAs was observed.

**Figure 2:**
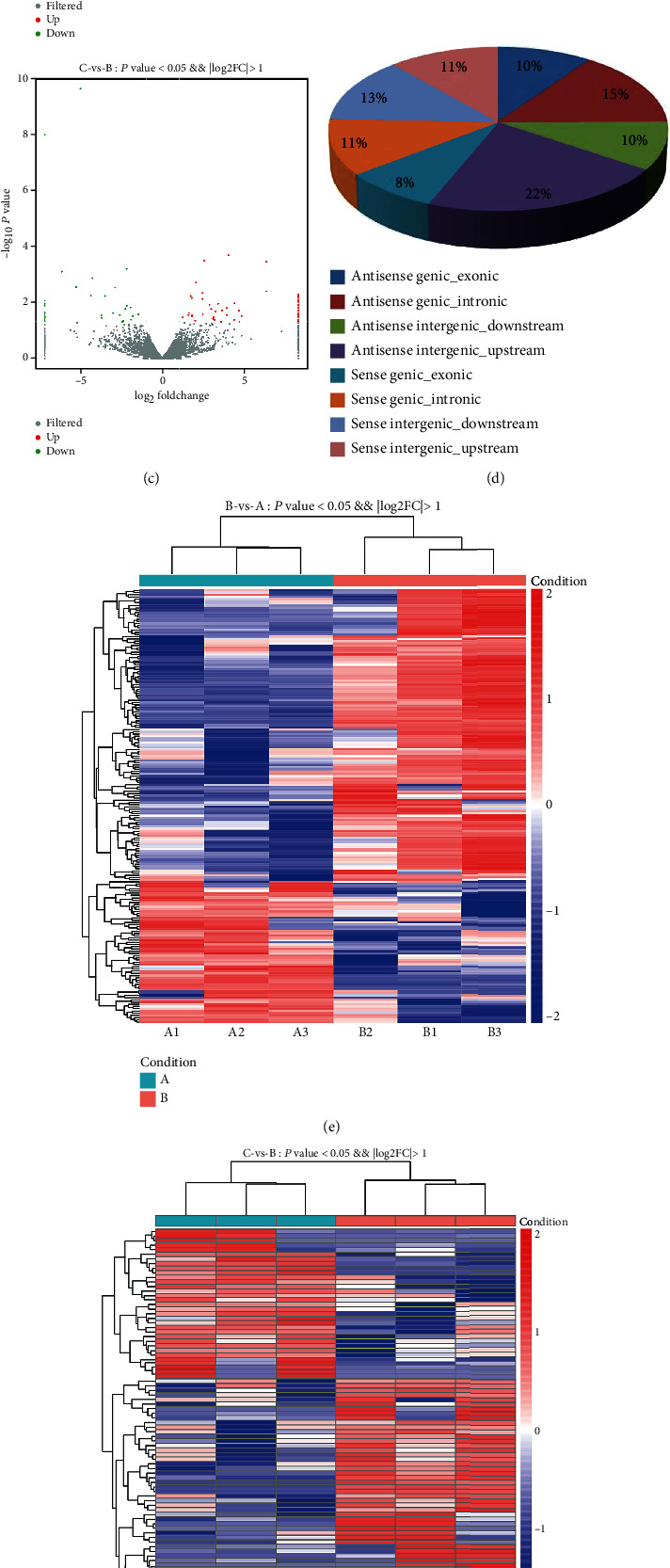
Differentially expressed lncRNAs in the lung tissues of rats in the three groups. (a) A box and whisker plot of lncRNAs showing the distributions of RNA intensities in all samples. (b) Heat map of correlation coefficients for all samples. (c) Volcano plots showing variation in the lncRNA expression. The vertical lines correspond to 2-fold change, and the horizontal line represents a *P* value of 0.05. (d) Type and proportion of lncRNAs. (e) Hierarchical clustering of all differentially expressed lncRNAs in the lung tissues of rats in groups A and B. (f) Hierarchical clustering of all differentially expressed lncRNAs in the lung tissues of rats in groups B and C. (g) Differentially expressed genes were analyzed using DEGseq software based on the FPKM method (≥2-fold change with *P* <0.05). The number of differentially expressed lncRNAs was observed.

**Figure 3:**
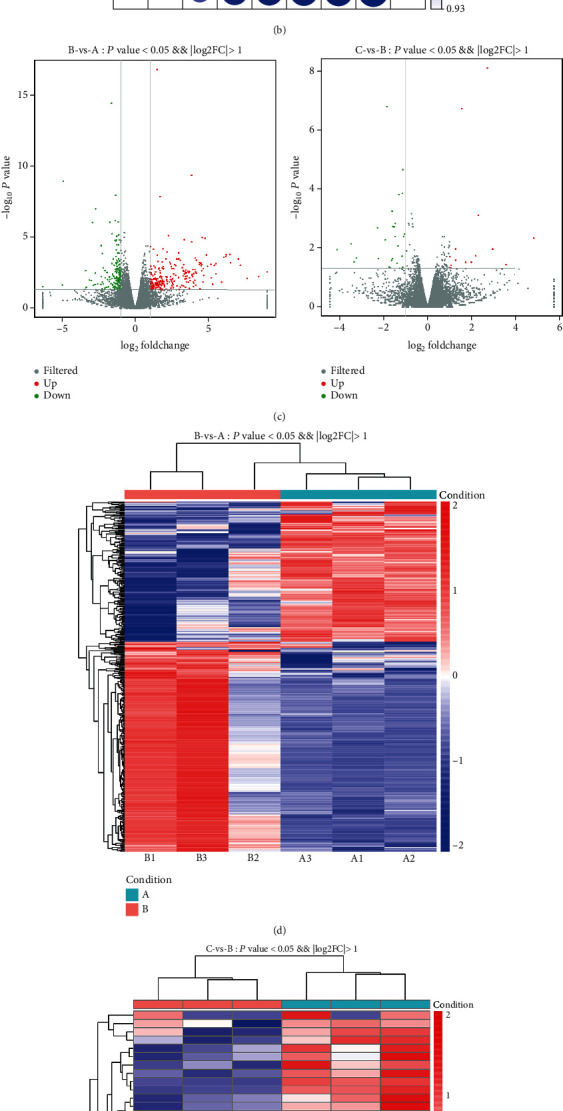
Differentially expressed mRNAs in the lung tissues of rats in the three groups. (a) A box and whisker plot of mRNAs showing the distributions of RNA intensities in all samples. (b) Heat map of correlation coefficients for all samples. (c) Volcano plots showing variation in the mRNA expression. The vertical lines correspond to 2-fold change, and the horizontal line represents a *P* value of 0.05. (d) Hierarchical clustering of all differentially expressed mRNAs in the lung tissues of rats in groups A and B. (e) Hierarchical clustering of all differentially expressed mRNAs in the lung tissues of rats in groups B and C. (f) Differentially expressed genes were analyzed using DEGseq software based on the FPKM method (≥2-fold change with *P* <0.05). The number of differentially expressed mRNAs was observed.

A total of 22,601 mRNAs, 10,187 lncRNAs, and 7,231 circRNAs were identified in the three groups. In order to compare the distributions of RNA intensities among all the samples, we used a box and whisker plot to visualize the distribution of each dataset. These plots showed no statistical difference in the circRNA, lncRNA, and mRNA distributions in the samples (Figures 1(a), 2(a), and 3(a). The correlation coefficients of the circRNA, lncRNA, and mRNA profiles among three biological replicates of the nine samples were 0.255–0.980, 0.936–0.988, and 0.931–0.997, respectively (Figures 1(b), 2(b), and 3(b). A total of 109 circRNAs, 393 lncRNAs, and 560 mRNAs were shown to be differentially expressed with a fold change of ≥2.0, *P* value of <0.05, and false discovery rate (FDR) of <0.05 in the three groups (Figures 1(c), 2(c), and 3(c), Tables S1–S9). According to the dysregulated circRNAs relation with protein-coding genes, they were divided into four categories—exonic (94.03%), intergenic (1.6%), intronic (1%), and antisense (3.37%) (Figure 1(d)). However, the majority (56.26%) of lncRNAs were antisense, whereas 24.31% were intergenic, 11.08% were intronic, and only 8.35% were exonic (Figure 2(d)). Compared with that observed in group A, 27 circRNAs, 133 lncRNAs, and 233 mRNA expressions were increased and 29 circRNAs, 65 lncRNAs, and 156 mRNA expressions were reduced in group B (Figures 1(e)–1(g), 2(e)–2(g), and 3(d)–3(f)). Compared with that observed in group B, 9 circRNAs, 55 lncRNAs, and 14 mRNAs were upregulated; and 9 circRNAs, 33 lncRNAs, and 27 mRNAs were downregulated in group C (Figures 1(e)–1(g), 2(e)–(g), and 3(d)–3(f)).

### 3.3. GO Analysis

**Figure 4:**
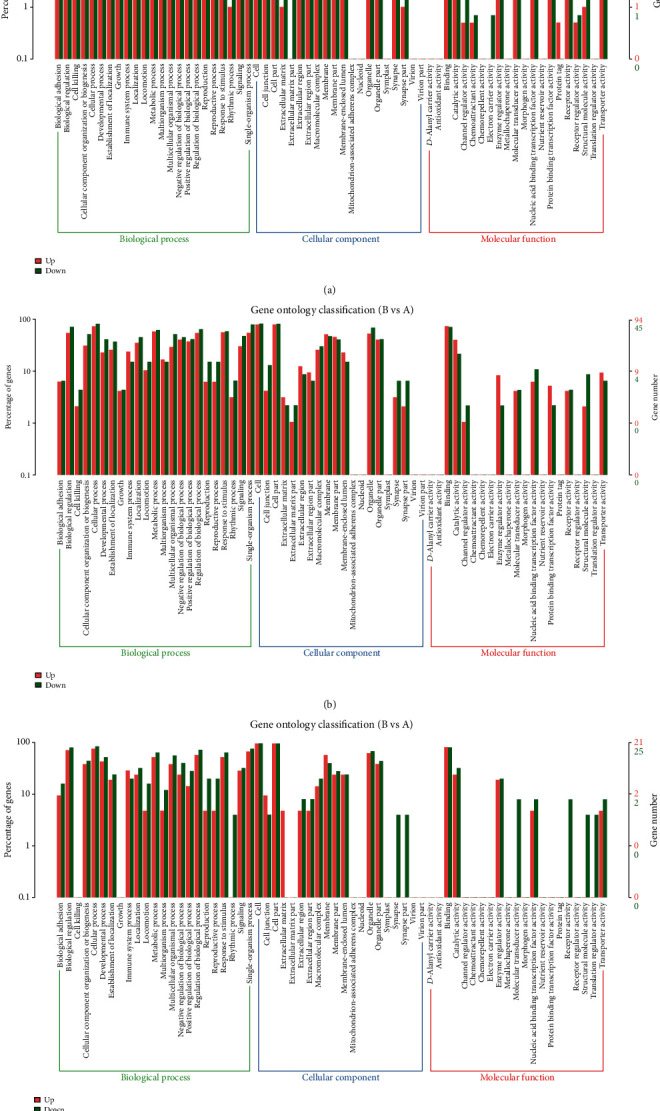
GO analyses of differentially expressed mRNAs, lncRNA targets, and circRNA genes between groups A and B. (a) Enrichment of biological processes, cellular components, and molecular functions in differentially expressed mRNAs. (b) Enrichment of biological processes, cellular components, and molecular functions in differentially expressed lncRNAs. (c) Enrichment of biological processes, cellular components, and molecular functions in differentially expressed circRNAs.

**Figure 5:**
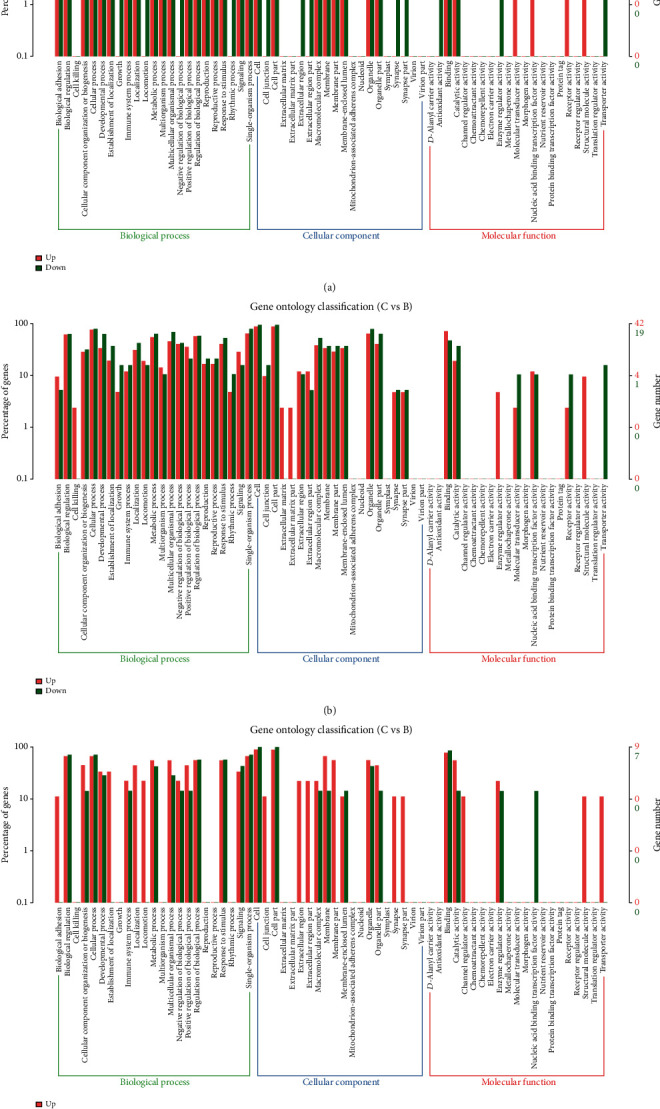
GO analyses of differentially expressed mRNAs, lncRNA targets, and circRNA genes between groups B and C. (a) Enrichment of biological processes, cellular components, and molecular functions in differentially expressed mRNAs. (b) Enrichment of biological processes, cellular components, and molecular functions in differentially expressed lncRNAs. (c) Enrichment of biological processes, cellular components, and molecular functions in differentially expressed circRNAs.

In order to investigate the functions of the abnormally expressed circRNAs, lncRNAs, and mRNAs, GO annotation enrichment analyses were carried out. GO analysis classified differently expressed genes on the basis of three aspects (biological processes (BP), cellular components (CC), and molecular functions (MF)). Of the genes with aberrant mRNA targets between groups A and B, 1,558 were related to BP, 273 with CC, and 732 with MF (Figure 4(a), Table S10). Of the genes with aberrant mRNA targets between groups B and C, 289 were related to BP, 51 with CC, and 68 with MF such as channel regulation (Figure 5(a), Table S13). Of the dysregulated lncRNAs between groups A and B, 913 were related to BP, 182 with CC, and 250 with MF (Figure 4(b), Table S11); of those between groups B and C, 435 were related to BP, 120 with CC, and 126 with MF (Figure 5(b), Table S14). Of the dysregulated circRNAs between groups A and B, 296 were associated with BP, 87 with CC, and 101 with MF (Figure 4(c), Table S12); of those between groups B and C, 113 were associated with BP, 49 with CC, and 57 with MF (Figure 5(c), Table S15). GO analysis showed that aberrant lncRNA targets are mainly associated with regulation of the interleukin-4-mediated signaling pathway, symbiont-containing vacuole membranes, and STAT family protein binding. Differentially expressed circRNA genes were found to mainly participate in immune system processes, lysosome activity, and enzyme inhibitor activity (Figures 4 and 5).

### 3.4. KEGG Pathway Analysis

**Figure 6:**
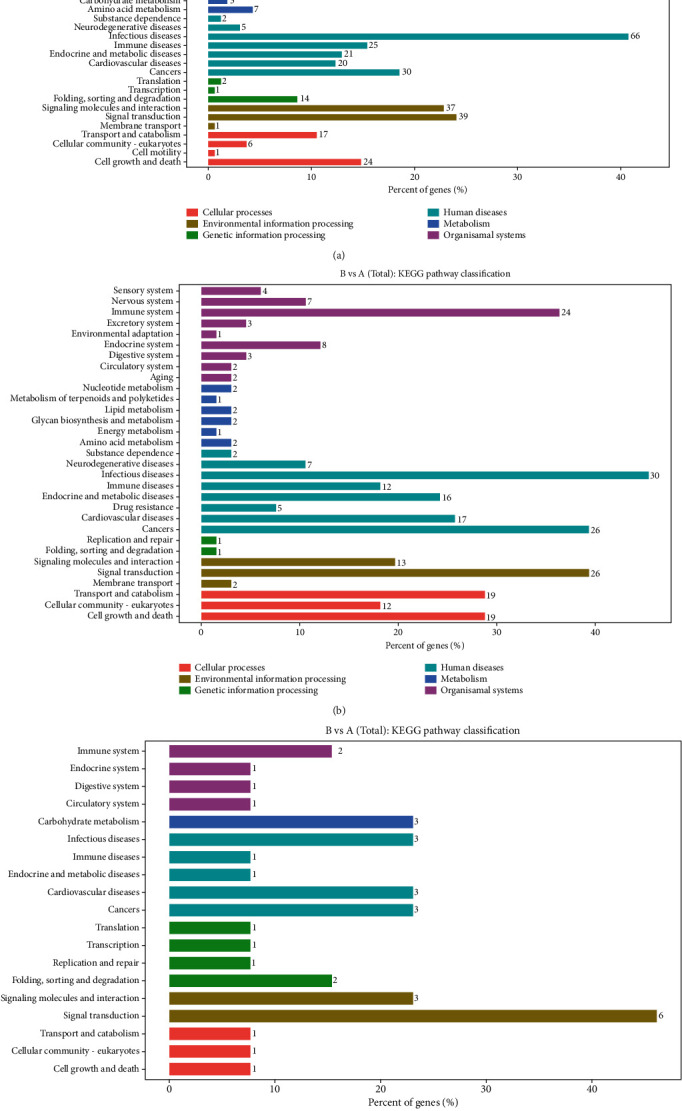
KEGG pathway classification of identified genes. The abscissa represents the annotated genes in the KEGG database; the ordinate represents categories in the KEGG database. (a) KEGG pathway classification of mRNAs that were differently expressed between groups A and B. (b) KEGG pathway classification of lncRNAs that were differently expressed between groups A and B. (c) KEGG pathway classification of circRNAs that were differently expressed between groups A and B.

**Figure 7:**
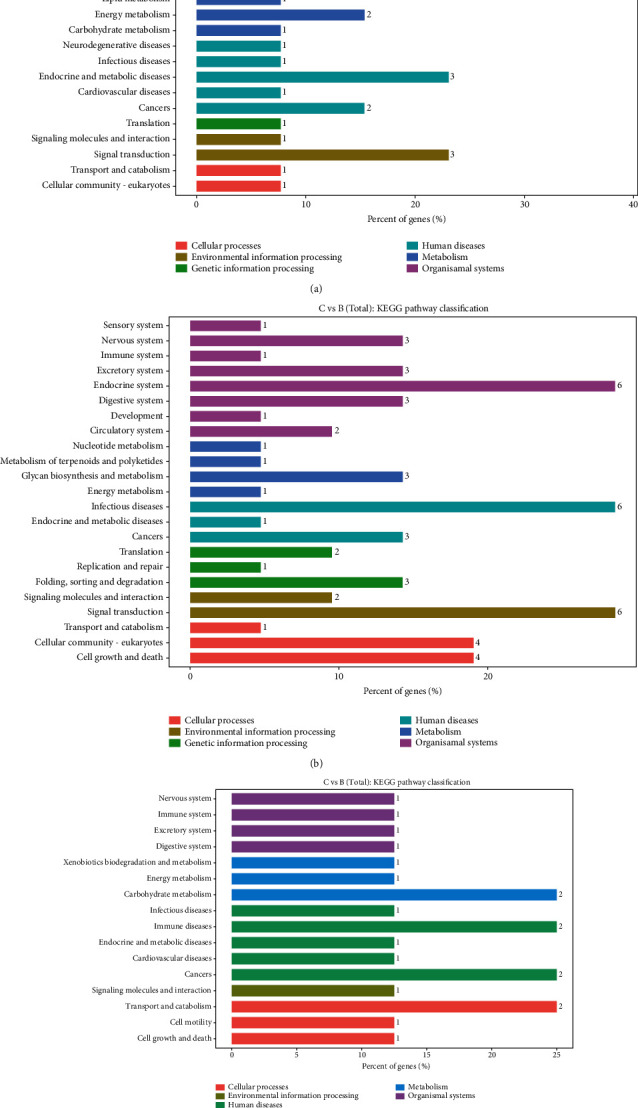
KEGG pathway classification of identified genes. The abscissa represents the annotated genes in the KEGG database; the ordinate represents categories in the KEGG database. (a) KEGG pathway classification of mRNAs that were differently expressed between groups B and C. (b) KEGG pathway classification of lncRNAs that were differently expressed between groups B and C. (c) KEGG pathway classification of circRNAs that were differently expressed between groups B and C.

To further explore the biological functions of the identified genes in the present study, obviously, enriched pathways were confirmed through comparing them to the KEGG database (Figures 6 and 7). The 462 differentially expressed mRNAs between groups A and B were annotated to 35 metabolic pathways. Among these pathways, the “immune system” included the most aberrant mRNAs (69), followed by “infectious diseases” (66), “signal transduction” (39), “signaling molecules and interaction” (37), and “cancers” (30) (Figure 6(a), Table S16). The 272 differentially expressed lncRNAs between groups A and B were annotated to 31 metabolic pathways. Among these pathways, “infectious diseases” included the most aberrant lncRNAs (30), followed by “cancers” (26), “signal transduction” (26), and “immune system” (24) (Figure 6(b), Table S17). The 36 differentially expressed circRNAs between groups A and B were annotated to 19 metabolic pathways. Among these pathways, “signal transduction” included the most aberrant circRNAs (6), followed by “carbohydrate metabolism” (3), “infectious diseases” (3), “cardiovascular diseases” (3), and “cancers” (3) (Figure 6(c), Table S18).

The 31 differently expressed mRNAs between groups B and C were annotated to 19 pathways. Most of these mRNAs were clustered in the “endocrine system” category (5), followed by “signal transduction” (3), “endocrine and metabolic diseases” (3), “environmental adaptation” (3), and “energy metabolism” (2) (Figure 7(a), Table S19). The 59 differently expressed lncRNAs between groups B and C were annotated to 23 metabolic pathways. Among these pathways, “signal transduction,” “infectious diseases,” and “endocrine system” included the most aberrant lncRNAs (6), followed by “cell growth and death” (4) and “cellular community: eukaryotes” (4) (Figure 7(b), Table S20). The 20 differently expressed circRNAs between groups B and C were annotated to 16 metabolic pathways. Among these pathways, “transport and catabolism,” “cancers,” “immune diseases,” and “carbohydrate metabolism” included the most aberrant lncRNAs (2) (Figure 7(c), Table S21).

### 3.5. Coexpression of circRNAs and miRNAs

**Figure 8:**
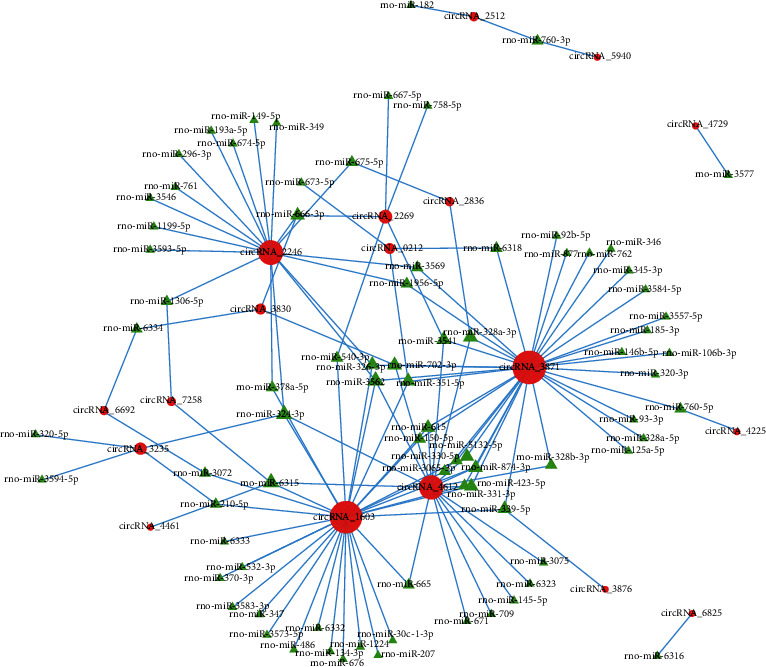
Construction of a circRNA–miRNA coexpression network for groups A and B. Red circle and green triangle represent circRNA and miRNA, respectively.

**Figure 9:**
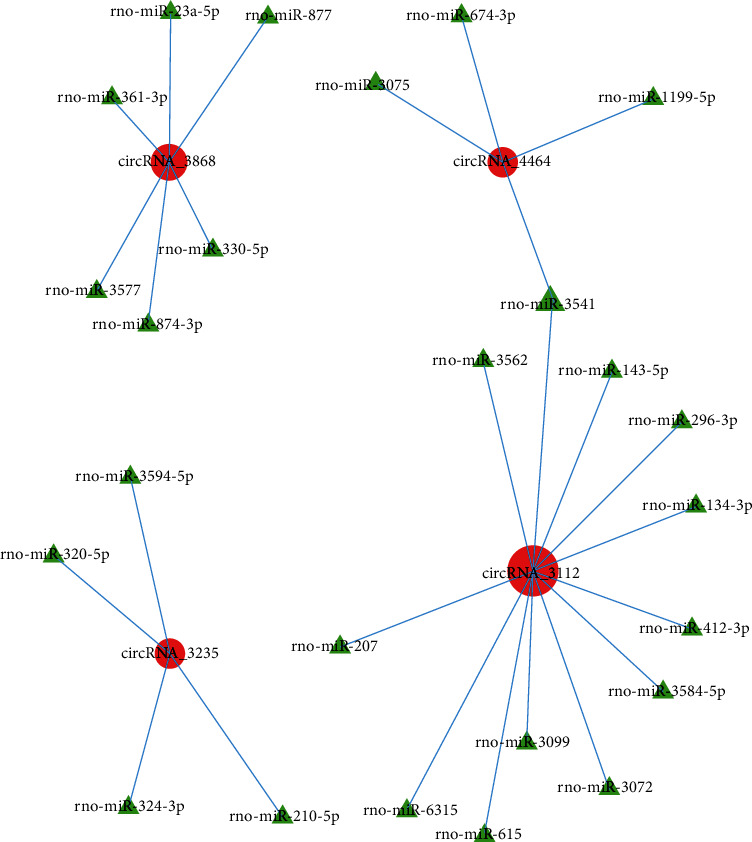
Construction of a circRNA–miRNA coexpression network for groups B and C. Red circle and green triangle represent circRNA and miRNA, respectively.

To investigate the underlying mechanisms of circRNA and phosgene-induced lung injury based on differentially expressed circRNA data, we used a database to predict target miRNAs interacting with circRNAs. The enrichment results of the total differences in circRNAs revealed that the top 300 miRNA–circRNA interaction pairs with small *P* values were extracted in order of the *P* value, and the *R* network software package was performed to establish a circRNA-targeted miRNA gene network map (Figures 8 and 9, Table S22). First, 18 significantly differentially expressed circRNAs were selected between groups A and B (Figure 8). Some of these circRNAs comprise multiple miRNA binding sites, whereas some share miRNA response elements. For example, circRNA-3871 comprises binding sites for miR-339-5p, miR-320-3p, miR-346, and miR-345-3p, and circRNA-2246 comprises binding sites for miR-149-5p, miR-296-3p, and miR-3593-5p. Both circRNA-3871 and circRNA-2246 target miR-1956-5p (Figure 8). Similarly, four significantly differentially expressed circRNAs were selected between groups B and C (Figure 9). Of these circRNAs, circRNA-3868 comprises binding sites for miR-877, miR-357, and miR-874-3p, and circRNA-3235 comprises binding sites for miR-210-5p, miR-3594-5p, and miR-320-5p. Both circRNA-4464 and circRNA-3112 target miR-3541 (Figure 9). These results suggest that circRNAs serve as efficient miRNA sponges in ALI.

## 4. Discussion

In the present study, we used transcriptome sequencing to compare the expression profiles of mRNAs, lncRNAs, and circRNAs in the lung tissues of rats in the air control, phosgene-exposed, and phosgene + MSC groups. Furthermore, we conducted GO and KEGG analyses and constructed regulation networks. Our study is aimed at elucidating the molecular mechanisms of phosgene-induced ALI after MSC treatment to identify biomarkers and new therapeutic targets for lung injury.

Previous studies on gene regulation have focused on protein-coding genes. However, in recent years, with the discovery of many ncRNAs, such as microRNAs, lncRNAs, and circRNAs, this view has changed [19]. The roles of lncRNAs in the progression and treatment of lung diseases have been reported. For example, MALAT1 was reportedly related to acute respiratory distress syndrome related to lung injury [20], downregulation of SNHG14 had protective effects against LPS-induced ALI [21], and CASC2 improved ALI by reducing lung epithelial cell apoptosis [22]. A potential relationship between lung injury and circRNAs has been demonstrated [18], revealing that circRNAs might have an important role in lung injury. In this study, we analyzed the abnormal expression profiles of lncRNAs, circRNAs, and mRNAs for the first time in phosgene-induced ALI after MSC treatment.

We determined the expression profiles of mRNAs, lncRNAs, and circRNAs using transcriptome sequencing. A total of 22,601 mRNAs, 10,187 lncRNAs, and 7,231 circRNAs were identified, and a total of 109 circRNAs, 393 lncRNAs, and 560 mRNAs were observed to be differentially expressed in the three groups with a fold change of ≥2.0, *P* value of <0.05, and FDR of <0.05. The majority (56.26%) of the lncRNAs were antisense, and approximately 94.03% of the circRNAs were exonic. Compared with that observed in group A, 27 circRNAs, 133 lncRNAs, and 233 mRNAs were increased, and 29 circRNAs, 65 lncRNAs, and 156 mRNAs were decreased in group B. Compared with that observed in group B, 9 circRNAs, 55 lncRNAs, and 14 mRNAs were upregulated, and 9 circRNAs, 33 lncRNAs, and 27 mRNAs were reduced in group C. In our study, we found that fatty acid-binding protein 4 (Fabp4) mRNA and lncRNA plasmacytoma variant translocation 1 (PVT1) were downregulated in group C than that in group B. Previous studies have shown that Fabp4 and PVT1 were reported to be participated in the immune response. For example, Fabp4 inhibitors suppress inflammation and oxidative stress in murine and cell models of acute lung injury [23], and suppression of Fabp4 protects against rhabdomyolysis-induced acute kidney injury [24]. In chronic obstructive pulmonary disease patients, the PVT1 expression positively correlated with the GOLD stage and levels of TNF-*α*, IL-6, IL-8, and IL-17 [25]; PVT1 exacerbates the inflammation and cell-barrier injury during asthma by regulating miR-149 [26]. These findings suggested that Fabp4 and PVT1 might play the role in the therapeutic potential of MSCs in phosgene-induced ALI, and we will research the potential role of them in the phosgene-induced ALI in the following research. The differentially expressed lncRNAs, mRNAs, and circRNA may play roles in phosgene-induced ALI and may prove to be important in the treatment of ALI using MSCs.

GO and KEGG analysis indicated that the main mechanisms of lung injury included single-organism processes, drug metabolism, and immune system processes. We also found that the lncRNA TCONS_00026162 (GO: 0060487) was associated with lung epithelial cell differentiation, TCONS_00026162 (GO: 0060441) was associated with epithelial tube branching involved in lung morphogenesis, TCONS_00026162 (GO: 0030324) was associated with lung development, and XR_001839103.1 and XR_593920.2 (GO: 0055114) were associated with the oxidation–reduction process. Meanwhile, circRNA_4627 (GO: 2000791 and GO: 0048286) was associated with the negative regulation of MSC proliferation involved in lung development and lung alveolus development, circRNA_5485 (GO: 0055114) was associated with the oxidation–reduction process, and circRNA_4178 (GO: 0002526) was associated with the acute inflammatory response. Lung epithelial cell differentiation has been reported to play key roles in various models of lung injury [27, 28]. Melittin exerts beneficial function on paraquat-induced lung injury by regulating oxidative stress and apoptosis [29]. Cordycepin suppresses LPS-caused ALI by preventing inflammation and oxidative stress [30]. Puerarin prevents LPS-induced ALI via inhibition of the inflammatory response [31]. These results suggest that the above mentioned lncRNAs and circRNAs may be involved in ALI.

Increasing evidence shows that natural endogenous circRNAs are inherently resistant to exonucleolytic RNA decay, and that they contain selectively conserved miRNA target sites. Therefore, circRNAs can function as efficient miRNA sponges, interacting with miRNA to regulate the gene expression [14, 32]. For example, the circRNA PVT1 facilitates osteosarcoma metastasis through regulation of the miR-526b/FOXC2 axis [33], and circRNA-33186 participated in the pathogenesis of osteoarthritis through functioning as a sponge of miR-127-5p [34]. In the present study, the potential target miRNAs were predicted, and the *R* network software package was used to establish a circRNA-targeted miRNA gene network map. circRNA-3871 contains binding sites for miR-339, miR-320-3p, miR-346, and miR-345-3p, and circRNA-2246 comprises binding sites for miR-149-5p, miR-296-3p, and miR-3593-5p. Compared with that observed in group A, the circRNA-3871 expression was upregulated in group B, and its target miRNA—miR-339—reportedly attenuates inflammation and inhibits pulmonary microvascular endothelial cell apoptosis in mice with severe acute pancreatitis-associated ALI [35]. Meanwhile, the circRNA-3235 expression was lower in group B than that in group C, and the expression of its target miR-320 was increased in ALI induced by cardiopulmonary bypass [36]. These findings suggest that circRNA-3871 functions as a miR-339 sponge and circRNA-3235 functions as a miR-320 sponge. Both these circRNAs may participate in phosgene-induced ALI progression and prove to be important in the treatment of ALI using MSCs. In our next study, we will verify this conjecture further.

In conclusion, we compared the expression profiles of mRNAs, lncRNAs, and circRNAs in the lung tissues of rats in three groups. Furthermore, we conducted GO and KEGG analyses and constructed coexpression networks. In addition, we used a database to predict target miRNAs interacting with circRNAs and the *R* network software package to establish a circRNA-targeted miRNA gene network map. Our study is aimed at elucidating the molecular mechanisms of phosgene-induced ALI after MSC treatment to identify biomarkers and new therapeutic targets for lung injury.

## Figures and Tables

**Table 1:** Summary of raw reads after quality control and mapping to the reference genome.

Sample	Raw reads	Clean reads	Clean reads rate	Q30	Mapped reads	Mapping rate
A 1	96.20 M	92.82 M	96.49%	93.95%	89.86 M	96.81%
A2	95.89 M	93.19 M	97.18%	94.62%	90.25 M	96.85%
A3	96.92 M	91.00 M	93.89%	91.55%	87.97 M	96.67%
P1	97.82 M	95.15 M	97.27%	94.86%	92.22 M	96.93%
P2	99.13 M	96.66 M	97.51%	94.81%	93.52 M	96.75%
P3	99.69 M	97.15 M	97.45%	94.88%	94.02 M	96.78%
PM1	100.89 M	98.35 M	97.48%	94.74%	95.21 M	96.80%
PM2	96.94 M	94.31 M	97.29%	94.72%	91.21 M	96.71%
PM3	94.86 M	90.64 M	95.55%	92.94%	87.48 M	96.51%

## Data Availability

We provide our data in the Supplementary Information files that we submit alongside our manuscript. The data used to support the findings of this study are available from the corresponding author upon request.

## Supplementary Materials

Supplementary 1 Table S1: differentially expressed mRNAs between the A and B group.

Supplementary 2 Table S2: differentially expressed lncRNAs between the A and B group.

Supplementary 3 Table S3: differentially expressed circRNAs between the A and B group.

Supplementary 4 Table S4: differentially expressed mRNAs between the B and C group.

Supplementary 5 Table S5: differentially expressed lncRNAs between the B and C group.

Supplementary 6 Table S6: differentially expressed circRNAs between the B and C group.

Supplementary 7 Table S7: differentially expressed mRNAs between A and C group.

Supplementary 8 Table S8: differentially expressed lncRNAs between the A and C group.

Supplementary 9 Table S9: differentially expressed circRNAs between the A and C group.

Supplementary 10 Table S10: GO with differentially expressed mRNAs between the A and B group.

Supplementary 11 Table S11: GO with differentially expressed lncRNAs between the A and B group.

Supplementary 12 Table S12: GO with differentially expressed circRNAs between the A and B group.

Supplementary 13 Table S13: GO with differentially expressed mRNAs between the B and C group.

Supplementary 14 Table S14: GO with differentially expressed lncRNAs between the B and C group.

Supplementary 15 Table S15: GO with differentially expressed circRNAs between the B and C group.

Supplementary 16 Table S16: KEGG with differentially expressed mRNAs between the A and B group.

Supplementary 17 Table S17: KEGG with differentially expressed lncRNAs between the A and B group.

Supplementary 18 Table S18: KEGG with differentially expressed circRNAs between the A and B group.

Supplementary 19 Table S19: KEGG with differentially expressed mRNAs between the B and C group.

Supplementary 20 Table S20: KEGG with differentially expressed lncRNAs between the B and C group.

Supplementary 21 Table S21: KEGG with differentially expressed circRNAs between the B and C group.

Supplementary 22 Table S22: circRNA-targeted miRNA.
